# Feasibility and acceptability of a culturally adapted medical nutrition therapy intervention for adults with type 2 diabetes in Benin: a single-arm mixed-method study

**DOI:** 10.3389/fnut.2026.1954177

**Published:** 2026-10-28

**Authors:** Halimatou Alaofè, Yasmine Degbelo, Edward John Bedrick, Alfred Acakpo, Lionel Medenou, John Ehiri

**Affiliations:** 1 Mel and Enid Zuckerman College of Public Health, The University of Arizona, Tucson, AZ, United States; 2 Faculté des Sciences de la Santé, Université d’Abomey-Calavi, Cotonou, Littoral, Benin; 3 Department of Epidemiology and Biostatistics, Mel and Enid Zuckerman College of Public Health, The University of Arizona, Tucson, AZ, United States

**Keywords:** diabetes self-management, feasibility and acceptability, low-resource settings, sub-Saharan Africa, type 2 diabetes culturally adapted medical nutrition therapy

## Abstract

**Introduction:**

Medical nutrition therapy (MNT) is a cornerstone of type 2 diabetes (T2D) management, but access to culturally appropriate nutrition care remains limited in many low-resource settings. We evaluated the feasibility and acceptability of OSanDiaBé, a culturally adapted MNT intervention for adults with T2D in Cotonou, Benin, West Africa.

**Methods:**

Guided by the Obesity-Related Behavioral Intervention Trials (ORBIT) framework, we conducted an ORBIT Phase IIa single-arm, pre-post mixed-methods feasibility study among adults aged 40–65 years with T2D. The intervention combined two individualized nutrition counseling sessions with eight group sessions delivered by trained dietitians and nutritionists in community settings over 12 weeks. Primary outcomes were recruitment, retention, attendance, intervention fidelity, and acceptability. Secondary exploratory outcomes included diabetes knowledge, diet quality, physical activity, diabetes self-efficacy, anthropometry, blood pressure, and glycated hemoglobin (HbA1c). Quantitative data were analyzed descriptively and using paired comparisons, while qualitative data were analyzed using reflexive thematic analysis.

**Results:**

Among 42 adults screened, 36 were enrolled (85.7%), and 30 completed the 12-week follow-up (83.3% retention). Mean attendance was 7.2 of 8 group sessions, and intervention fidelity exceeded 85%. The prespecified acceptability progression criterion was met, with a mean overall satisfaction rating of 4.87 ± 0.35, exceeding the prespecified threshold of ≥4.0. Exploratory improvements were observed in overall diabetes knowledge (50.0–83.3%), diet quality (+9.29 points), regular physical activity participation (26.7–86.7%), and diabetes self-efficacy (+15.45 points). Mean HbA1c decreased by 0.58 percentage points (95% CI −1.12 to −0.04), with modest reductions also observed in body weight and body mass index. Qualitative findings identified culturally tailored educational materials, practical tools, peer support, and community-based delivery as important factors supporting participant engagement and intervention acceptability.

**Discussion:**

OSanDiaBé was feasible to implement and highly acceptable among adults with T2D in urban Benin. These findings support progression to a pilot randomized controlled trial to further evaluate the intervention and generate preliminary estimates to inform subsequent effectiveness, implementation, economic, and scalability research.

## 1 Introduction

Type 2 diabetes (T2D) is a growing global public health challenge, with the greatest increases occurring in low- and middle-income countries (LMICs). In 2024, an estimated 589 million adults worldwide were living with diabetes, nearly 80% of whom resided in LMICs (1). Sub-Saharan Africa (SSA) is projected to experience the fastest growth, with the number of adults living with diabetes expected to more than double by 2045 (1–3). Rapid urbanization, nutrition transition, characterized by shifts from traditional diets toward more energy-dense, highly processed foods and changing dietary patterns, and increasingly sedentary lifestyles are accelerating the burden of non-communicable diseases across the region (2, 3).

Benin reflects these regional trends. According to the International Diabetes Federation, approximately 467,000 adults were living with diabetes in Benin in 2024, with projections approaching 1.1 million by 2050 (1). National and regional studies have likewise documented increasing diabetes prevalence, particularly in urban settings (4). Despite this growing burden, access to comprehensive diabetes care remains limited. Structured diabetes self-management education, multidisciplinary chronic disease management, and specialized nutrition services are inconsistently available, contributing to poor glycemic control, diabetes-related complications, and premature mortality (2, 3).

Medical nutrition therapy (MNT) is a cornerstone of evidence-based T2D management and is recommended by major international clinical practice guidelines (5, 6). Beyond pharmacological treatment, individualized MNT delivered by trained nutrition professionals can improve glycemic control, dietary adherence, cardiometabolic risk factors, and quality of life while potentially reducing long-term healthcare costs (7–10). Consequently, expanding equitable access to high-quality nutrition care has become a priority for improving diabetes outcomes worldwide. However, most evidence supporting MNT has been generated in high-income countries, where healthcare systems, food environments, literacy levels, and availability of trained nutrition professionals differ substantially from those in LMICs (9).

Implementing MNT within many African health systems presents important structural and contextual challenges. These include shortages of trained dietitians and nutritionists, limited integration of nutrition services into routine diabetes care, financial barriers affecting food choices, low nutrition literacy, and the central role of traditional dietary practices in everyday life (11–14). Furthermore, nutrition recommendations developed in high-income settings often fail to reflect local food systems, household decision-making, affordability constraints, and sociocultural determinants of eating behaviors in SSA (15, 16). Consequently, although MNT is recognized as an essential component of diabetes care, relatively little evidence exists regarding how to deliver culturally appropriate, feasible, and sustainable MNT interventions within routine care in resource-constrained settings.

Emerging evidence suggests that culturally adapted nutrition interventions can improve acceptability, engagement, and self-management by aligning dietary recommendations with local foods, cultural practices, and community contexts (17–19). Interventions incorporating culturally familiar foods, family involvement, peer support, and community-based delivery have demonstrated promising behavioral outcomes among underserved populations (17, 18). Nevertheless, evidence from SSA remains limited. Few studies have evaluated culturally adapted MNT interventions that have been systematically developed, tailored to local contexts, and assessed using implementation-focused feasibility outcomes before randomized evaluation. This represents an important gap in both the clinical nutrition and implementation science literature.

Recognizing that successful implementation requires more than demonstrating efficacy, the Obesity-Related Behavioral Intervention Trials (ORBIT) framework emphasizes the systematic development, refinement, and preliminary testing of behavioral interventions before larger-scale efficacy and effectiveness trials (20, 21). Guided by this framework, we developed Objectif Santé Diabète Bénin (OSanDiaBé), a culturally adapted MNT intervention designed specifically for adults living with T2D in Benin. The intervention was informed by extensive formative mixed-methods research exploring dietary practices, sociocultural influences, family dynamics, and barriers to diabetes self-management in Benin (20, 22–24). It integrates individualized nutrition counseling, group-based experiential learning, culturally adapted educational materials, locally appropriate meal-planning strategies, and behavior-change techniques to promote sustainable dietary self-management within the local food environment.

The development of the OSanDiaBé intervention has been reported previously through its ORBIT intervention-development protocol (20). The present study represents the subsequent ORBIT Phase IIa proof-of-concept feasibility study, evaluating implementation of the intervention in an urban, resource-constrained setting in Benin. The primary objective was to determine whether prespecified progression criteria for recruitment, retention, attendance, intervention fidelity, and participant acceptability could be achieved to support progression to a pilot randomized controlled trial. Secondary exploratory objectives were to characterize changes in behavioral, psychosocial, and clinical outcomes and generate preliminary estimates to inform subsequent controlled evaluation of the intervention.

## 2 Materials and methods

### 2.1 Study design and setting

This ORBIT Phase IIa proof-of-concept study used a single-arm, pre-post mixed-methods feasibility design to evaluate the feasibility, acceptability, and implementation fidelity of the culturally adapted OSanDiaBé MNT intervention among adults with T2D in Cotonou, Benin (20, 21).

The study was conducted in Cotonou, the economic capital of Benin, a low-income West African country experiencing rapid urbanization and an increasing burden of T2D and other non-communicable diseases (1–3, 25, 26). Participants were recruited from outpatient diabetes clinics within secondary-level healthcare facilities providing routine diabetes care. Specialized nutrition and dietetic services remain limited in Benin, and structured diabetes self-management education and culturally adapted MNT are not routinely integrated into clinical practice (2, 11).

The intervention was delivered within existing healthcare and community structures to assess its feasibility under locally relevant implementation conditions and inform subsequent evaluation.

### 2.2 Participants and recruitment

Participants were recruited between September and October 2024 from outpatient diabetes clinics in Cotonou through collaboration with healthcare providers involved in routine diabetes care. Eligible participants were adults aged 40–65 years with a clinical diagnosis of T2D for at least 1 year, suboptimal glycemic control (HbA1c ≥ 7%), and the ability to participate in group-based educational sessions. Participants were also required to have at least one family member or household contact available to support diabetes self-management activities. Individuals were excluded if they were pregnant or breastfeeding, unable to provide informed consent, had severe cognitive impairment, advanced diabetes-related complications, or serious medical conditions that could make participation unsafe or substantially interfere with intervention participation, such as unstable cardiovascular disease, advanced kidney disease or renal failure, active cancer requiring intensive treatment, or severe mobility-limiting illness.

Consistent with the ORBIT framework, participants were recruited from individuals who had participated in the Phase Ib intervention refinement study to support iterative optimization of the intervention before pilot randomized evaluation (20, 21). Eligible individuals received verbal and written study information in French, with translation into local languages when needed, and provided written informed consent before enrollment.

In keeping with recommendations for ORBIT Phase IIa and feasibility studies, a sample of 36 participants was considered sufficient to evaluate recruitment, retention, intervention adherence, fidelity, acceptability, and other feasibility parameters rather than intervention effectiveness (27, 28). Family members were invited to participate in selected intervention sessions to strengthen social support and facilitate household-level lifestyle changes (29).

### 2.3 Intervention development and description

The development of the OSanDiaBé intervention has been described previously (20). Briefly, the intervention was developed using the ORBIT framework through iterative formative research, stakeholder engagement, and intervention refinement during Phases Ia and Ib (20–24). Formative mixed-methods studies identified key determinants of diabetes self-management in Benin, including dietary practices, sociocultural influences, family dynamics, affordability constraints, and barriers to implementing medical nutrition therapy (22–24). Guided by Social Cognitive Theory and the Socioecological Model (30, 31), these findings informed the development of a culturally adapted MNT intervention tailored to the local food environment.

Cultural tailoring was informed by qualitative interviews, stakeholder consultations, and participatory intervention-refinement activities with adults living with T2D, family members, dietitians, and healthcare providers (20, 22–24). Findings from these activities were translated into specific intervention adaptations, including use of familiar locally available foods and traditional meals in dietary counseling; portion-estimation methods based on commonly used household utensils and hand measures; culturally adapted recipes and meal-planning examples that considered affordability and local food availability; low-literacy visual materials; accommodation of local languages when clarification was needed; and incorporation of family and peer support to reflect household and social influences on food practices. Intervention content and delivery strategies were iteratively reviewed and refined with participants and local stakeholders during ORBIT Phases Ia and Ib before feasibility testing (20, 22–24).

During ORBIT Phase IIa, the intervention was delivered over 12 weeks and combined two individualized home-based nutrition counseling sessions (45–60 min each) with eight community-based group sessions (90–120 min each), facilitated by 6 trained dietitians and nutritionists. Group sessions were delivered in cohorts of approximately 10–12 participants and followed a progressive behavioral structure organized around three phases: Understand, Act, and Maintain.

The intervention addressed diabetes self-management through culturally adapted nutrition education, healthy eating using locally available foods, portion control, meal planning, healthy food purchasing and preparation, physical activity, goal setting, relapse prevention, and family and peer support. Educational strategies included experiential learning, cooking demonstrations, interactive discussions, problem-solving activities, and culturally adapted visual materials (22–24, 32).

Participants received a culturally tailored recipe booklet (Mes repas santé OSanDiaBé), developed using locally available foods and linear goal programming to optimize nutritional quality while maintaining affordability and cultural acceptability (24, 33). Food affordability was explicitly considered during intervention development and delivery because formative research identified household financial constraints as an important barrier to dietary adherence. Dietary recommendations therefore emphasized locally available foods, lower-cost alternatives, meal planning within household budgets, and adaptation of traditional meals rather than reliance on specialized diabetes foods. The culturally tailored recipe booklet was developed to balance nutritional adequacy, local availability, cultural acceptability, and affordability. Additional educational tools included healthy plate visuals, portion-estimation techniques using household utensils and hand measurements, participant workbooks, and low-literacy educational materials adapted to the local context (16, 22).

Individual counseling sessions emphasized dietary assessment, collaborative goal setting, identification of barriers to adherence, and personalized action planning using the Nutrition Care Process and Problem–Etiology–Signs/Symptoms (PES) framework (6, 34). Group sessions promoted peer support, experiential skill-building, and structured physical activity adapted for adults with T2D (29, 32). Participants also received ongoing reinforcement through a moderated WhatsApp group between sessions.

The intervention incorporated evidence-based behavior change techniques, including goal setting, self-monitoring, action planning, problem-solving, feedback, and mobilization of social support (35, 36). Before intervention delivery, the 6 dietitians and nutritionists completed standardized facilitator training covering the OSanDiaBé MNT protocol, use of the facilitator manual and participant workbook, culturally adapted food and portion guidance, counseling and behavior-change techniques, group facilitation, and intervention fidelity procedures. Training incorporated didactic instruction, practical demonstrations, role-play, and standardized practice using intervention materials. Facilitators subsequently used the same session guides, locally adapted food examples, recipe materials, portion-estimation tools, and fidelity checklists across intervention cohorts. Ongoing supervision and session observations were used to reinforce standardized delivery (20, 37). Participants received transportation reimbursement and small participation incentives to support attendance. A detailed description of the intervention is provided in Figure 1 and Supplementary Table 1.

**Figure 1:**
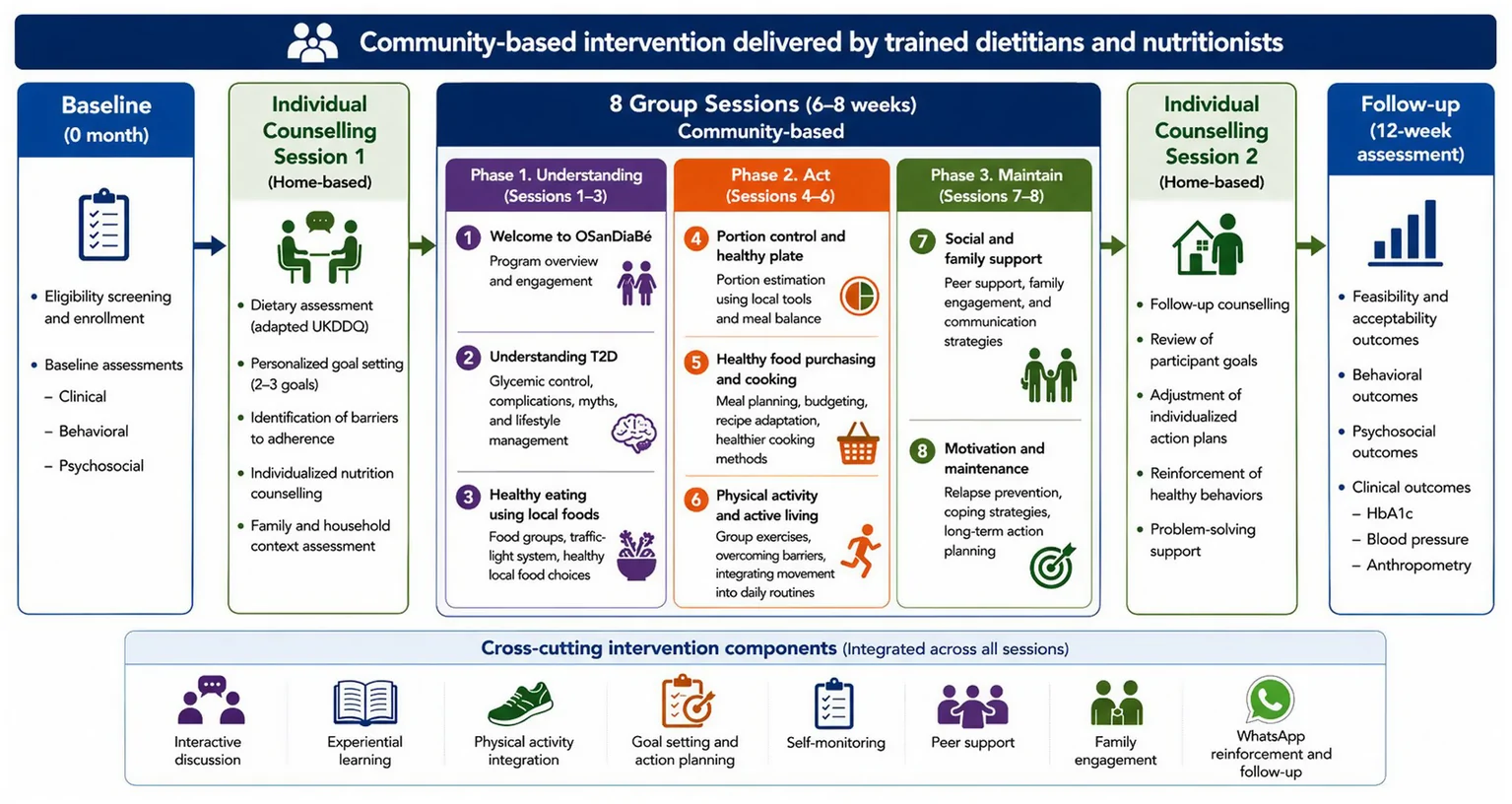
Structure and delivery of the OSanDiaBé intervention during the ORBIT Phase IIa feasibility study. HbA1c, glycated hemoglobin; OSanDiaBé, Objectif Santé Diabète Bénin; T2D, Type 2 Diabetes; UKDDQ, United Kingdom Diabetes and Diet Questionnaire.

### 2.4 Outcomes and measures

The primary outcomes were the feasibility and acceptability of the OSanDiaBé intervention. Feasibility outcomes included recruitment, retention, session attendance, completeness of outcome data, intervention adherence, implementation fidelity, and adverse events (27, 28, 37).

Prespecified progression criteria were established to determine whether the intervention was suitable for progression to a pilot randomized controlled trial. Feasibility was considered acceptable if the study achieved: (1) recruitment of at least 80% of eligible individuals screened; (2) retention of ≥80% at the 12-week follow-up; (3) mean attendance of ≥75% of group sessions; (4) intervention fidelity of ≥80% of planned intervention components; and (5) a mean participant rating of ≥4.0 on the 5-point overall satisfaction item. These criteria were informed by published recommendations for feasibility and pilot studies (27, 37).

Implementation fidelity was assessed using standardized facilitator checklists, session observations, and facilitator logs documenting delivery of planned intervention components according to the intervention manual (37). Acceptability was evaluated using a post-intervention process evaluation questionnaire administered during the final group session. The questionnaire included Likert-scale items and open-ended questions assessing participant satisfaction, cultural relevance, perceived usefulness, appropriateness of intervention components, and recommendations for improvement (37, 38).

Secondary exploratory outcomes included changes in diabetes knowledge, dietary behaviors, physical activity, diabetes self-efficacy, family functioning, anthropometric measures, blood pressure, and glycated hemoglobin (HbA1c). These outcomes were selected to evaluate hypothesized pathways through which culturally adapted MNT may improve diabetes self-management and glycemic control (6, 20, 30). Baseline and follow-up assessments were conducted at enrollment and 12 weeks by research assistants who received standardized training in study procedures, questionnaire administration, anthropometric and blood pressure measurements, data recording, and participant confidentiality before data collection.

Behavioral and psychosocial outcomes were assessed using validated instruments adapted for the local context. Dietary adherence was measured using an adapted version of the United Kingdom Diabetes and Diet Questionnaire (UKDDQ), with higher scores indicating healthier dietary behaviors (39). Diabetes knowledge was assessed using the Audit of Diabetes Knowledge (ADKnowl) questionnaire (40). Family functioning was evaluated using the General Functioning subscale of the McMaster Family Assessment Device (FAD-GF12), with scores ≤2.0 indicating healthy family functioning (41). Diabetes self-efficacy was assessed using the Diabetes Self-Efficacy Scale (DSES), with higher scores indicating greater confidence in diabetes self-management (42).

Anthropometric and clinical outcomes were measured using standardized protocols. Body weight and height were measured using calibrated equipment, and body mass index (BMI) was calculated according to World Health Organization criteria (43). Waist circumference was measured using a non-elastic measuring tape at the level of the umbilicus. Blood pressure was measured after at least five minutes of seated rest using a calibrated sphygmomanometer, and the average of valid measurements was used for analysis. Glycemic control was assessed by measuring HbA1c with a HemoCue HbA1c analyzer following standardized quality-control procedures (5, 6).

Baseline sociodemographic and clinical characteristics included age, sex, marital status, education, employment status, household income, diabetes duration, treatment regimen, and selected health behaviors. Sex was self-reported by participants at enrollment and categorized as female or male. Gender identity was not separately assessed because the study’s original sociodemographic questionnaire collected sex but did not include a distinct measure of gender identity.

### 2.5 Process evaluation and qualitative methods

A mixed-methods process evaluation, informed by the Medical Research Council guidance for complex interventions, was conducted to assess intervention acceptability, implementation fidelity, participant engagement, and contextual factors influencing intervention delivery and participation (37, 38).

Quantitative process evaluation data were collected using structured post-intervention questionnaires assessing participant satisfaction, cultural relevance, engagement with intervention activities, facilitator support, and perceived intervention benefits. Open-ended questions explored participants’ experiences, barriers to participation, and recommendations for intervention refinement. Facilitator perspectives were obtained through structured debriefing discussions and implementation notes documenting participant engagement, logistical challenges, fidelity to intervention procedures, and recommendations for improving delivery.

Qualitative data from participant feedback and facilitator discussions were analyzed using reflexive thematic analysis as described by Braun and Clarke (44). Two members of the research team engaged with the qualitative data through iterative coding and reflexive discussion. Coding and candidate themes were reviewed collaboratively to explore alternative interpretations, deepen engagement with the data, and refine the developing thematic structure. Themes were subsequently considered across participant and facilitator perspectives to provide a richer contextual understanding of intervention implementation (44, 45). Qualitative findings were integrated with quantitative feasibility outcomes to provide a comprehensive understanding of intervention implementation and to inform refinement of the intervention for future pilot randomized evaluation.

### 2.6 Data analysis

Descriptive statistics were used to summarize participant characteristics, feasibility outcomes, process-evaluation findings, and exploratory behavioral and clinical outcomes. Continuous variables were summarized using means and standard deviations, while categorical variables were summarized using frequencies and percentages. Individual 5-point Likert items from the post-intervention process evaluation were treated as ordinal measures and summarized descriptively using medians and first and third quartiles (Q1–Q3). The prespecified acceptability progression criterion was based on the mean rating for the overall satisfaction item, with a threshold of ≥4.0.

Consistent with the exploratory objectives of this ORBIT Phase IIa feasibility study, analyses focused primarily on estimating feasibility parameters and preliminary changes in behavioral and clinical outcomes with 95% confidence intervals rather than testing intervention effectiveness (27, 28). Because normality assumptions were not consistently met, within-participant changes in continuous outcomes were assessed using the Wilcoxon signed-rank test, and paired binary outcomes were analyzed using exact McNemar tests. Analyses of change from baseline to 12 weeks were restricted to participants with complete paired data, and missing outcome data were not imputed. *p*-values are reported as exploratory and should be interpreted cautiously. No adjustment for multiple comparisons was performed because secondary outcome analyses were exploratory and intended to generate preliminary estimates for future trials. Quantitative analyses were conducted using Stata (StataCorp, College Station, TX, United States).

Qualitative data were managed using NVivo (QSR International, Melbourne, Australia) and analyzed using reflexive thematic analysis (44, 45). Quantitative and qualitative findings were integrated during interpretation to contextualize feasibility outcomes, identify implementation barriers and facilitators, and inform refinement of the intervention before pilot randomized evaluation.

## 3 Results

### 3.1 Participant flow and feasibility outcomes

Participant flow is summarized in Figure 2. Of the 52 adults with T2D identified as potentially eligible from the Phase Ib intervention-refinement cohort, 42 were contacted and screened for eligibility. Of those screened, 36 (85.7%; 95% CI 71.5–94.6%) met the eligibility criteria, completed the baseline assessment, and were enrolled in the ORBIT Phase IIa feasibility study. Six individuals were not enrolled because they did not meet the eligibility criteria (*n* = 2), declined participation (*n* = 2), or were unable to participate because of illness or travel (*n* = 2).

**Figure 2:**
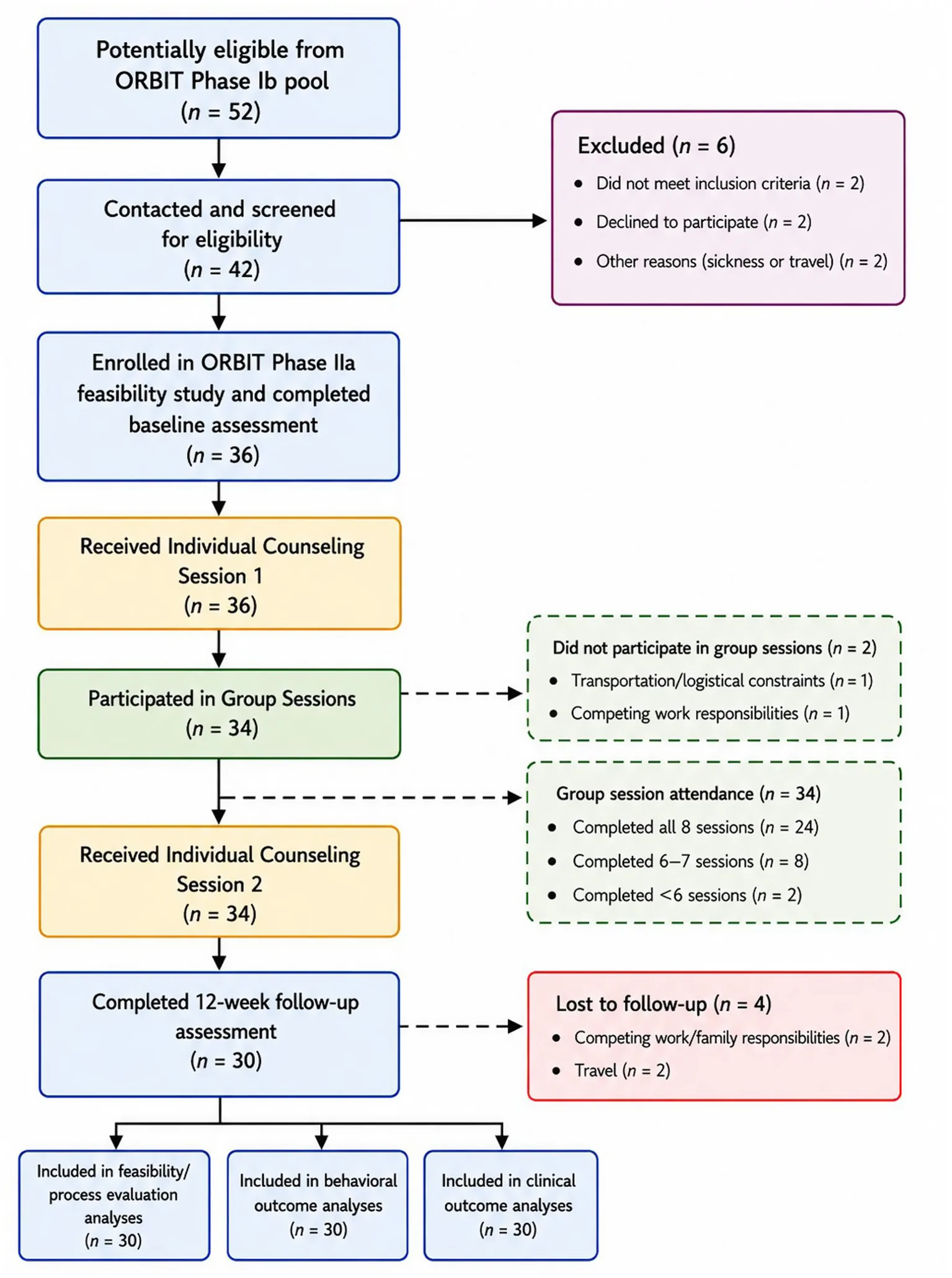
CONSORT-style participant flow diagram for the OSanDiaBé ORBIT Phase IIa feasibility study. Flow diagram illustrating participant screening, enrollment, intervention participation, retention, follow-up completion, and inclusion in feasibility and exploratory outcome analyses during implementation of the culturally adapted OSanDiaBé intervention among adults living with type 2 diabetes in Benin.

The prespecified recruitment and retention progression criteria were met. Recruitment exceeded the ≥80% threshold, with 85.7% of screened individuals enrolled. At 12 weeks, 30 of the 36 enrolled participants completed follow-up assessments, corresponding to a retention rate of 83.3% (95% CI 65.3–94.4%). Four participants were lost to follow-up because of competing work or family responsibilities (*n* = 2) or travel (*n* = 2). Of the six participants who did not complete the 12-week assessment, two did not participate in the group sessions because of transportation or logistical constraints (*n* = 1) or competing work responsibilities (*n* = 1), and four were subsequently lost to follow-up because of competing work or family responsibilities (*n* = 2) or travel (*n* = 2).

All 36 participants received the first individualized nutrition counseling session, and 34 completed the second individualized counseling session. Of the 36 participants, 34 participated in the group sessions, while 2 did not participate because of transportation or logistical constraints (*n* = 1) or competing work responsibilities (*n* = 1). Among the 34 participants who attended group sessions, 24 completed all 8 sessions, 8 completed 6–7 sessions, and 2 completed fewer than 6 sessions. Mean attendance was 7.2 of the 8 planned group sessions (95% CI 6.7–7.7), corresponding to an attendance rate of 90% and exceeding the prespecified ≥75% attendance threshold.

The intervention was delivered according to the planned 12-week protocol. Implementation fidelity exceeded the prespecified threshold of ≥80%, with facilitators delivering more than 85% of planned intervention components based on standardized fidelity checklists, session observations, and facilitator logs. Individual counseling sessions demonstrated high adherence to the Nutrition Care Process, including completion of dietary assessments, collaborative goal setting, and follow-up action planning.

Participant engagement was high throughout the intervention. Participants actively engaged in educational discussions, physical activity sessions, and individualized goal-setting activities, and the moderated WhatsApp group was used to reinforce key messages and support engagement between sessions. No intervention-related adverse events were reported.

### 3.2 Baseline participant characteristics

Baseline sociodemographic and clinical characteristics are presented in Table 1. Participants had a mean age of 50.4 ± 8.4 years and had lived with T2D for a mean of 11.2 ± 7.8 years. Most participants were female (55.6%), married (66.7%), and self-employed (61.1%). Educational attainment was relatively high, with 52.8% having completed secondary education and 36.1% having attained university education. Participants lived in relatively large households, with a mean household size of 6.0 ± 2.9 individuals.

**Table 1:** Baseline sociodemographic and clinical characteristics of study participants (*n* = 36).

Characteristics	n (%) or Mean ± SD
Sociodemographic characteristics	
Age, years	50.44 ± 8.41
Diabetes duration, years	11.21 ± 7.83
Family size	6.03 ± 2.90
Sex	
Female	20 (55.56)
Male	16 (44.44)
Marital status	
Married	24 (66.67)
Single	10 (27.78)
Widowed	2 (5.56)
Religion	
Christian	20 (55.56)
Muslim	6 (16.67)
Other^a^	10 (27.78)
Education level	
No formal education	1 (2.78)
Primary education	3 (8.33)
Secondary education	19 (52.78)
University education	13 (36.11)
Employment status	
Government employment	3 (8.33)
Non-governmental employment	7 (19.44)
Independent worker	22 (61.11)
Retired	4 (11.11)
Perceived household income adequacy	
Does not cover basic needs	7 (19.44)
Covers only usual expenses	15 (41.67)
Covers usual expenses and emergencies	5 (13.89)
Allows savings or investment	9 (25.00)
Diabetes treatment regimen	
Lifestyle only	1 (2.78)
Lifestyle + antidiabetic medication	19 (52.78)
Lifestyle + antidiabetic medication + insulin	14 (38.89)
Lifestyle + insulin	2 (5.56)
Clinical characteristics	
Body mass index (BMI), kg/m^2^	28.93 ± 4.69
Normal weight	8 (22.22)
Overweight	14 (38.89)
Obesity	14 (38.89)
Waist circumference, cm	95.81 ± 7.81
Systolic blood pressure, mmHg	133.42 ± 19.72
Diastolic blood pressure, mmHg	91.27 ± 15.11
HbA1c, %	7.67 ± 0.64

BMI, body mass index; HbA1c, glycated hemoglobin; SD, standard deviation.

^a^Other religions included Céleste, Protestant/Evangelical, and Eckankar.

Most participants reported financial constraints, with 61.1% indicating that household income covered only routine expenses or was insufficient to meet basic household needs. Regarding diabetes management, 52.8% were treated with antidiabetic medications in combination with lifestyle modification, while 38.9% received antidiabetic medications together with insulin therapy.

At baseline, participants exhibited a high cardiometabolic risk profile. Mean BMI was 28.9 ± 4.7 kg/m^2^, and 77.8% of participants were classified as overweight or obese. Mean waist circumference was 95.8 ± 7.8 cm, mean blood pressure was 133.4 ± 19.7 mmHg systolic and 91.3 ± 15.1 mmHg diastolic, and mean HbA1c was 7.67 ± 0.64%, indicating suboptimal glycemic control at study entry.

### 3.3 Intervention acceptability and process evaluation

Process-evaluation findings indicated high levels of participant satisfaction, cultural acceptability, and engagement with the OSanDiaBé intervention (Table 2). Participants rated the cultural relevance of intervention content and materials highly, with a median score of 5 (Q1–Q3: 4–5), and reported favorable experiences with community-based delivery, local-language accommodation, individualized nutrition counseling, group education, and practical intervention activities.

**Table 2:** Process evaluation outcomes of the OSanDiaBé intervention among study participants who completed the 12-week follow-up (*n* = 30).

Statements	Median (Q1–Q3)
Cultural relevance and contextual appropriateness	
The intervention content and educational materials were culturally relevant and appropriate.	5 (4–5)
Program facilitators were respectful of my culture, beliefs, and food practices.	5 (5–5)
Delivery of the program in French did not negatively affect my participation or understanding.	4 (3–5)
Facilitator willingness to translate into local languages improved my engagement in the program.	5 (4–5)
Co-facilitation of educational sessions was acceptable and beneficial.	4 (4–5)
Community-based delivery of the intervention facilitated my participation.	5 (5–5)
Physical activity sessions were culturally appropriate and adapted to participant abilities.	5 (5–5)
Intervention delivery and participant engagement	
Group sessions facilitated my engagement in the program.	5 (4–5)
Individual counseling sessions facilitated my engagement in the program.	5 (5–5)
Program facilitators were knowledgeable about diabetes and nutrition.	5 (4–5)
Program facilitators were supportive and approachable.	5 (5–5)
Physical activity sessions were enjoyable and appropriate for participants with different abilities.	5 (5–5)
The structure of each session was clear and easy to follow.	5 (4–5)
The frequency of sessions was appropriate.	5 (5–5)
Session duration (90–120 min) was appropriate.	4 (4–5)
Discussing progress, barriers, and weekly action plans facilitated my engagement in the program.	5 (4–5)
Healthy plate demonstrations and portion-control activities were understandable and practical.	5 (5–5)
WhatsApp group discussions were useful and supportive.	5 (4–5)
I felt supported and included as a member of the group.	5 (4–5)
Perceived benefits of the intervention	
I gained valuable knowledge that I did not previously have.	5 (5–5)
I learned new skills related to physical activity.	5 (5–5)
I learned new skills related to healthy eating and meal planning.	5 (5–5)
I feel better physically and emotionally.	5 (4–5)
I have greater confidence in managing my diabetes.	5 (4–5)
I am more active and engaged with my family.	3 (2–4)
I regularly use skills and concepts learned during the program.	5 (4–5)
Overall satisfaction with the intervention	
I was satisfied with the OSanDiaBé intervention.	5 (5–5)
My participation in the program was enjoyable.	5 (5–5)
I would recommend this program to other adults living with T2D.	5 (5–5)

OSanDiaBé, Objectif Santé Diabète Bénin; SD, standard deviation; T2D, type 2 diabetes.

Responses were assessed using a 5-point Likert scale ranging from 1 (“strongly disagree”) to 5 (“strongly agree”) and are presented as median and first and third quartiles (Q1–Q3). The prespecified acceptability progression criterion was based on the mean rating for the overall satisfaction item (“I was satisfied with the OSanDiaBé intervention”); the observed mean ± SD was 4.87 ± 0.35, exceeding the prespecified threshold of ≥4.0.

The prespecified acceptability progression criterion was met, with a mean overall satisfaction rating of 4.87 ± 0.35, exceeding the prespecified threshold of ≥4.0. Overall satisfaction, enjoyment, and willingness to recommend the intervention to other adults with T2D each had a median score of 5 (Q1–Q3: 5–5). Qualitative findings provided additional context regarding participants’ experiences and factors influencing intervention acceptability, engagement, and implementation (Section 3.7).

### 3.4 Changes in diabetes knowledge and behavioral outcomes

Participants demonstrated substantial improvements in diabetes-related knowledge between baseline and 12 weeks post-intervention (Table 3). Overall diabetes knowledge increased from 50.0% at baseline to 83.3% at follow-up (*p* < 0.001). Significant gains were also observed in diet and food knowledge, treatment knowledge, foot-care knowledge, physical activity knowledge, knowledge of complication prevention, and hypoglycemia management. The largest improvement was observed in treatment knowledge, which increased from 23.3% at baseline to 93.3% at follow-up (+70.0 percentage points; *p* < 0.001). Improvements in sick-day management and smoking/alcohol-related knowledge did not reach statistical significance.

**Table 3:** Changes in diabetes knowledge, behavioral outcomes, psychosocial measures, and clinical outcomes from baseline to 12 weeks among participants who completed the intervention (*n* = 30).

Variables	Baseline mean ± SD or *n* (%)	Week 12 mean ± SD or *n* (%)	Change from baseline (Δ)	*p*-value
Diabetes knowledge domains				
Overall diabetes knowledge	15 (50.00)	25 (83.33)	+10 (33.33)	<0.001
Diet and food knowledge	16 (53.33)	26 (86.67)	+10 (33.33)	0.0039
Treatment knowledge	7 (23.33)	28 (93.33)	+21 (70.00)	<0.001
Sick-day management knowledge	17 (56.67)	25 (83.33)	+8 (26.67)	0.1573
Foot-care knowledge	12 (40.00)	24 (80.00)	+12 (40.00)	0.0030
Physical activity knowledge	16 (53.33)	23 (76.67)	+7 (23.33)	0.0196
Smoking/alcohol-related knowledge	17 (56.67)	24 (80.00)	+7 (23.33)	0.0522
Knowledge of complication prevention	18 (60.00)	28 (93.33)	+10 (33.33)	0.0039
Hypoglycemia knowledge	15 (50.00)	28 (93.33)	+13 (43.33)	0.0030
Behavioral and psychosocial outcomes				
Regular physical activity participation, *n* (%)	8 (26.67)	26 (86.67)	+18 (60.00)	<0.001
Diet quality score	69.07 ± 8.52	78.36 ± 10.03	+9.29 ± 9.00	<0.001
Diabetes self-efficacy score	50.34 ± 11.65	65.79 ± 7.32	+15.45 ± 10.90	<0.001
Healthy family functioning (FAD-GF12 ≤ 2.0), *n* (%)	16 (53.33)	19 (63.33)	+3 (10.00)	0.5315
Anthropometric and clinical outcomes				
Weight, kg	79.04 ± 12.88	78.25 ± 12.80	−0.80 ± 2.09	0.0259
Body mass index (BMI), kg/m^2^	28.80 ± 4.36	28.50 ± 4.25	−0.29 ± 0.75	0.0274
Waist circumference, cm	95.60 ± 10.95	94.80 ± 12.07	−0.80 ± 4.44	0.3576
Systolic blood pressure, mmHg	132.90 ± 20.21	128.86 ± 21.52	−4.00 ± 19.33	0.0763
Diastolic blood pressure, mmHg	88.62 ± 16.29	83.69 ± 14.21	−4.93 ± 12.38	0.0695
HbA1c, %	7.53 ± 1.74	6.95 ± 1.17	−0.58 ± 1.45	0.0403

BMI, body mass index; FAD-GF12, General Functioning subscale of the McMaster Family Assessment Device; HbA1c, glycated hemoglobin; SD, standard deviation.

Values are presented as mean ± standard deviation (SD) or n (%). Baseline values are restricted to participants with complete paired baseline and 12-week data (*n* = 30) and may therefore differ from baseline values reported for the full enrolled sample (*n* = 36) in Table 1. Changes from baseline to 12 weeks are presented descriptively as mean differences for continuous variables and percentage-point changes for categorical variables. *p*-values for continuous outcomes were obtained using Wilcoxon signed-rank tests and therefore do not represent tests of the reported mean differences. *p*-values for paired categorical outcomes were obtained using exact McNemar tests. All analyses were exploratory.

Behavioral outcomes also changed over the intervention period. The proportion of participants reporting regular physical activity increased from 26.7% at baseline to 86.7% at 12 weeks (+60.0 percentage points; *p* < 0.001). Mean diet quality scores increased from 69.07 ± 8.52 to 78.36 ± 10.03, corresponding to a mean increase of 9.29 points (95% CI 5.93–12.65; *p* < 0.001).

Supplementary Figure 1 illustrates changes in dietary behaviors from baseline to 12 weeks. Participants reported healthier dietary practices across multiple domains, including more regular meal consumption, healthier food purchasing, increased vegetable intake, reduced consumption of processed foods, and healthier snacking behaviors.

### 3.5 Psychosocial outcomes

Diabetes self-efficacy improved substantially over the intervention period, increasing by 15.45 points (95% CI 11.38–19.52), from 50.34 ± 11.65 at baseline to 65.79 ± 7.32 at 12 weeks (*p* < 0.001) (Table 3).

Healthy family functioning also improved modestly over the intervention period. The proportion of participants classified as having healthy family functioning increased from 53.3% at baseline to 63.3% at follow-up (+10.0 percentage points), although this change did not reach statistical significance (*p* = 0.5315).

Qualitative findings provided additional context for these psychosocial findings, including participants’ experiences of peer support, family engagement, and group cohesion (Section 3.7).

### 3.6 Anthropometric and clinical outcomes

Exploratory analyses showed changes in several anthropometric and clinical outcomes over the 12-week intervention period (Table 3). Mean body weight decreased from 79.04 ± 12.88 kg at baseline to 78.25 ± 12.80 kg at follow-up, corresponding to a mean reduction of 0.80 kg (95% CI −1.58 to −0.02; *p* = 0.0259). Similarly, mean BMI decreased from 28.80 ± 4.36 kg/m^2^ to 28.50 ± 4.25 kg/m^2^, corresponding to a mean reduction of 0.29 kg/m^2^ (95% CI −0.57 to −0.01; *p* = 0.0274).

Among participants with complete paired data, mean HbA1c decreased from 7.53 ± 1.74% at baseline to 6.95 ± 1.17% at 12 weeks, corresponding to a mean within-participant reduction of 0.58 percentage points (95% CI −1.12 to −0.04; *p* = 0.0403). Reductions were also observed in waist circumference and systolic and diastolic blood pressure, although these changes were not statistically significant.

### 3.7 Qualitative process evaluation findings

Reflexive thematic analysis identified five overarching themes describing participants’ and facilitators’ experiences with the OSanDiaBé intervention: (1) cultural relevance and practical applicability, (2) peer support and group cohesion, (3) increased confidence and self-management capacity, (4) implementation barriers and challenges, and (5) intervention fidelity and implementation observations. These themes complemented the quantitative process evaluation findings by providing additional context regarding intervention acceptability, participant engagement, and implementation.

#### 3.7.1 Cultural relevance and practical applicability

Most participants emphasized the importance of culturally adapted dietary guidance that incorporated locally available foods and familiar eating practices. Practical demonstrations, healthy plate activities, and recipe adaptation sessions were frequently described as particularly useful for translating nutrition recommendations into everyday practice. Participants also appreciated the flexibility to receive educational content in local languages, which improved comprehension and facilitated participation.

One participant explained:

*“The foods discussed were foods we already eat at home, so it was easier to apply the recommendations.”*

#### 3.7.2 Peer support and group cohesion

Participants consistently described the group sessions as a source of encouragement, shared learning, and accountability. Hearing the experiences of others living with T2D normalized common challenges and increased motivation to adopt and maintain healthier behaviors.

One participant stated:

*“We encouraged each other during the sessions and learned from each other’s experiences.”*

Facilitators similarly described strong group cohesion and active participation during educational discussions and physical activity sessions.

#### 3.7.3 Increased confidence and self-management capacity

Participants described increased confidence in meal planning, portion control, and diabetes self-management following the intervention. Some participants described applying newly acquired skills within their households and involving family members in dietary changes. Participants particularly highlighted improvements in understanding portion sizes, adopting healthier cooking methods, and maintaining regular meal schedules, findings that complemented the observed improvements in diabetes self-efficacy.

#### 3.7.4 Implementation barriers and challenges

Despite the overall positive experiences, participants identified several barriers that affected participation and long-term adherence, including transportation constraints, competing work responsibilities, financial limitations, and difficulties maintaining lifestyle changes in social settings.

Facilitators also noted variability in participants’ literacy levels and occasional scheduling challenges during group sessions. Nevertheless, they reported that the community-based delivery model and flexible facilitation strategies helped maintain participant engagement throughout the intervention.

#### 3.7.5 Intervention fidelity and implementation observations

Facilitators reported that the structured facilitator manual, standardized MNT protocol, participant workbook, and ongoing supervision supported consistent intervention delivery while allowing flexibility to address participants’ individual needs. Home visits, family engagement, and WhatsApp-based follow-up were viewed as particularly valuable for reinforcing behavior change and sustaining participant engagement between sessions. 

One facilitator reflected:

*“Participants became increasingly confident over time. By the later sessions, they were openly sharing strategies and encouraging one another to make healthier food choices.”*

Minor adaptations during implementation primarily involved clarifying concepts in local languages, tailoring examples to participants’ daily experiences, and adjusting session schedules to maximize attendance. Facilitators also suggested simplifying selected carbohydrate-counting activities and reorganizing some nutrition education content to further improve participant comprehension. These adaptations were implemented to facilitate intervention delivery while preserving the intervention’s core components.

## 4 Discussion

This ORBIT Phase IIa feasibility study demonstrated that the culturally adapted OSanDiaBé medical nutrition therapy (MNT) intervention was feasible to implement, highly acceptable, and delivered with high fidelity among adults living with T2D in an urban, resource-constrained setting in Benin. All prespecified feasibility progression criteria were met, including recruitment, retention, attendance, intervention fidelity, and participant satisfaction, supporting progression to a pilot randomized controlled trial. Participants demonstrated high engagement with both individualized counseling and group-based intervention components, while qualitative findings from participants and facilitators provided additional context regarding intervention acceptability and implementation.

These findings contribute to the limited evidence on culturally adapted MNT interventions in sub-Saharan Africa and support the feasibility of delivering contextually relevant diabetes self-management support within community and outpatient care settings. From a clinical nutrition perspective, the findings demonstrate that structured, culturally adapted MNT can be delivered by trained dietitians and nutritionists within community settings in an urban, resource-constrained African context. By combining individualized nutrition counseling with group education, family engagement, culturally appropriate educational materials, and practical skill-building activities, the intervention addressed locally identified barriers to dietary self-management. High participant satisfaction, strong attendance, and favorable qualitative findings further support the acceptability and cultural appropriateness of the intervention in this setting. Participants particularly valued familiar local foods, practical meal-planning activities, healthy plate demonstrations, peer interaction, and communication in local languages. These findings are consistent with previous evidence suggesting that culturally adapted nutrition interventions may support engagement and adherence among underserved populations (17–19).

### 4.1 Interpretation and implications

The intervention emphasized experiential learning, individualized nutrition counseling, and practical skill-building to support dietary self-management. Individual counseling enabled facilitators to address personalized challenges related to food affordability, household dynamics, and dietary practices, while group sessions provided opportunities for social support, shared learning, and accountability. Participants and facilitators also highlighted the value of home visits, family engagement, and WhatsApp-based follow-up for reinforcing intervention content between sessions. These findings are consistent with previous studies suggesting that peer support, family involvement, and low-cost digital communication strategies may strengthen diabetes self-management interventions in resource-constrained settings (17, 18, 29, 46, 47).

Exploratory analyses showed changes in diabetes-related knowledge, diet quality, physical activity participation, diabetes self-efficacy, body weight, BMI, and HbA1c over the 12-week study period. These findings should be interpreted cautiously given the single-arm design, modest sample size, and short follow-up and should be regarded as exploratory signals rather than estimates of intervention effectiveness. The observed mean reduction in HbA1c of 0.58 percentage points (95% CI −1.12 to −0.04) represents a noteworthy exploratory signal; however, this change cannot be attributed to the intervention and requires confirmation in a controlled study (7–10).

The findings also highlight the importance of culturally responsive implementation approaches within low-resource health systems. Facilitators reported that standardized manuals, structured session guides, participant workbooks, and ongoing supervision supported consistent intervention delivery while allowing flexibility to address participants’ individual needs. Facilitator recommendations, including simplifying carbohydrate-counting activities and restructuring selected nutrition education content, provide clear priorities for refining the intervention before evaluation in a pilot randomized controlled trial. These iterative refinements are consistent with the ORBIT framework’s emphasis on progressive intervention development and optimization before large-scale effectiveness testing (20, 21).

The feasibility and acceptability of OSanDiaBé also have implications for efforts to strengthen equitable diabetes care in sub-Saharan Africa. In many LMICs, access to MNT remains limited because of workforce shortages, financial constraints, and limited integration of nutrition services into routine diabetes care (2, 11, 14). The present findings suggest that community-based, culturally adapted MNT delivered by trained nutrition professionals within existing healthcare and community structures warrants further evaluation as a potential approach for expanding access to diabetes self-management support in resource-constrained settings.

### 4.2 Strengths and limitations

This study has several strengths. The intervention was developed through a systematic, theory-informed, and culturally grounded process that integrated formative research, stakeholder engagement, and iterative refinement. The use of the ORBIT framework provided a structured pathway for intervention development, feasibility testing, and preparation for subsequent randomized evaluation. The mixed-methods design integrated quantitative feasibility indicators with qualitative process evaluation findings, providing a comprehensive understanding of intervention implementation and participant experiences. The study also addresses an important evidence gap regarding culturally adapted MNT delivered by trained dietitians and nutritionists in sub-Saharan Africa. The mixed-methods design integrated quantitative feasibility indicators with qualitative process evaluation findings, providing a comprehensive understanding of intervention implementation and participant experiences (48).

Several limitations should be acknowledged. First, the single-arm pre-post design without a comparison group precludes attribution of the observed behavioral, psychosocial, and clinical changes to the intervention, as secular trends, regression to the mean, or other unmeasured factors may have contributed. Accordingly, secondary outcomes should be interpreted as exploratory signals rather than estimates of intervention effectiveness. Second, although the relatively small sample was appropriate for assessing feasibility, it limited the precision of exploratory clinical outcome estimates. Third, the 12-week follow-up period did not allow assessment of whether observed changes in dietary behaviors, self-management, and clinical outcomes were sustained over time.

Participants were recruited from urban outpatient clinics in Cotonou and had previously participated in the Phase Ib intervention-refinement study. They may therefore have been more motivated, familiar with intervention activities, or engaged in diabetes self-management than the broader population of adults with T2D. This may have contributed to the high attendance, retention, and acceptability observed and may limit generalizability to rural populations, individuals with lower healthcare engagement, and other sociocultural settings in Benin. In addition, gender identity was not assessed separately from sex, limiting our ability to examine potential gender-related differences in intervention experiences or outcomes. Detailed standardized data on individual comorbid conditions were also not collected at baseline beyond the clinical characteristics and eligibility-related conditions specified in the study protocol, precluding examination of whether comorbidity burden influenced dietary requirements, participation, adherence, or acceptability.

Finally, several behavioral outcomes relied on self-report and may have been affected by recall or social desirability bias, potentially influencing the magnitude of the observed behavioral changes. Although affordability was incorporated into intervention design, formal household food-expenditure and intervention cost-effectiveness data were not collected. Consequently, the economic affordability and cost-effectiveness of OSanDiaBé cannot be determined from this Phase IIa study. Subsequent controlled evaluations should include longer-term follow-up, systematic assessment of comorbidities, and economic evaluation.

Despite these limitations, the study provides evidence supporting the feasibility and acceptability of the culturally adapted OSanDiaBé MNT intervention in this urban Beninese setting. The findings support progression to a pilot randomized controlled trial to further evaluate the intervention and generate preliminary estimates to inform subsequent research on clinical effectiveness, implementation outcomes, economic evaluation, and scalability across diverse healthcare and community contexts in Benin.

## 5 Conclusion

The culturally adapted OSanDiaBé medical nutrition therapy intervention was feasible to implement and highly acceptable among adults living with T2D in urban Benin. High participant engagement, intervention fidelity, and favorable process-evaluation findings support the feasibility of delivering the intervention through trained dietitians and nutritionists within community-based settings. Exploratory changes were observed in diabetes knowledge, self-management behaviors, psychosocial outcomes, anthropometric measures, and HbA1c; however, these findings cannot be attributed to the intervention and require confirmation in a controlled study. The findings support progression to a pilot randomized controlled trial to further evaluate OSanDiaBé and inform subsequent research on effectiveness, implementation, economic outcomes, and scalability.

## Data Availability

The original contributions presented in the study are included in the article/Supplementary material, further inquiries can be directed to the corresponding author/s.
