# Supplementary material for: Feasibility and acceptability of a culturally adapted medical nutrition therapy intervention for adults with type 2 diabetes in Benin: a single-arm mixed-method study
Source: Front Nutr. 2026 Oct 28;13:1954177. doi: 10.3389/fnut.2026.1954177 (PMC13752178; doi:10.3389/fnut.2026.1954177)
Supplement: Supplementary file 2 [file Table_1.docx]

**Supplementary Table 1.** Structure and content of the OSanDiaBé intervention

| Phase | Session | Theme | Objective | Key components |
| --- | --- | --- | --- | --- |
| Understanding *(Build understanding and awareness)* | 1 | Welcome to OSanDiaBé | Introduce the intervention and initiate motivation for behavior change | Program overview; introduction to diabetes self-management; relationship between diet, physical activity, and glycemic control; participant expectations; collaborative goal setting; group engagement activities |
|  | 2 | Understanding type 2 diabetes | Build foundational knowledge of T2D and self-management | Causes, symptoms, and complications of T2D; glycaemia and HbA1c; myths and misconceptions; role of nutrition, physical activity, and medication adherence; discussion of local beliefs and practices |
|  | 3 | Choosing foods to control blood sugar | Improve understanding of healthy food choices within local food systems | Food groups using adapted traffic-light classification; role of vegetables, carbohydrates, proteins, and fats; identifying healthier traditional foods; reading food labels where applicable; dietary awareness and self-monitoring |
| Act *(Develop practical self-management skills)* | 4 | Portion control and meal balance | Improve portion regulation and meal planning skills | Portion estimation using local household tools (hand, bowl, spoon); healthy plate model; meal timing; glycemic impact of portion sizes; culturally adapted meal examples and demonstrations |
|  | 5 | Healthy purchasing and cooking | Improve food preparation practices and household food choices | Meal planning; healthy food purchasing strategies within limited budgets; reducing oil, sugar, and salt; adapting traditional recipes; healthier cooking methods; recipe demonstrations using local foods |
|  | 6 | Physical activity and active living | Increase daily physical activity and reduce sedentary behavior | Types and benefits of physical activity for T2D; overcoming barriers to exercise; culturally appropriate group exercises; stretching and low-impact activities; integrating movement into daily routines |
| Maintain *(Strengthen maintenance and long-term adherence)* | 7 | Building support systems | Strengthen family, peer, and healthcare support for diabetes self-management | Role of family members, peers, and healthcare providers; communication strategies; managing social situations and food-related pressures; social support for maintaining healthy behaviors |
|  | 8 | Maintaining motivation and preventing relapse | Support long-term maintenance of healthy lifestyle behaviors | Review of progress and achievements; coping strategies; relapse prevention; problem-solving techniques; reinforcing healthy habits; individualized long-term goal setting and action planning |

**Abbreviations**: HbA1c, glycated hemoglobin; OSanDiaBé, Objectif Santé Diabète Bénin; T2D, type 2 diabetes.
